# How to Maximize Children's Involvement in Non-therapeutic Research—Lessons Learnt From EFFECTOR

**DOI:** 10.3389/fped.2020.00047

**Published:** 2020-02-14

**Authors:** Karolien Van De Maele, Roland Devlieger, Inge Gies

**Affiliations:** ^1^Division of Pediatric Endocrinology, KidZ Health Castle, University Hospital Brussels, Jette, Belgium; ^2^Research unit Organ Systems, Department of Development and Regeneration, Catholic University of Leuven, Leuven, Belgium; ^3^Research Unit GRON, Free University of Brussels, Jette, Belgium; ^4^Department of Obstetrics and Gynecology, University Hospital of Leuven, Leuven, Belgium

**Keywords:** clinical research, non-therapeutic research, children, pediatrics, ethics in research with children

## Abstract

**Background:** Children are vulnerable study subjects, especially in non-therapeutic research. Nowadays more attention is paid to the children's voice in both decision-making on participation and their experience of clinical research procedures.

**Methods:** We share our experiences from a long-term, cross-sectional, non-therapeutic follow-up study in the offspring of mothers who participated in scientific research during their pregnancy.

**Results:** During the data collection process, different strategies were developed to achieve a satisfactory participation rate with a focus on the involvement of the children. All study documents and measurements were assembled into a superhero framework. This theme is flexible and attracts children of a wide age-span. In order to inform the children before the study visit, a visually attractive assent was created as well as a superhero video. During the study visit, a sticker diploma was used with similar visuals from the assent. The toddlers received a superhero-cape. The children were involved in the decision-making process during the whole process.

**Discussion and conclusion:** From our experience during the EFFECTOR data collection process, parents and their children can be motivated to participate in a long-term, non-therapeutic, follow-up study when child friendly and adequate communication is used. Framing in a superhero theme is simple and suitable for children of a wide age-span.

## Introduction

Children are considered to be vulnerable research study subjects and in recent years, more attention has been paid to their own voice in the process of consent and participation in scientific research (1, 2). Different taskforces were established to promote scientific research in children and to support scientists to do this in an ethical way (3–6). The European Pediatric Investigations Plans, for example, resulted in a positive impact on pediatric drug development (7). Non-drug studies could benefit from a similar approach.

A specific entity in scientific research are the non-therapeutic studies, where no direct therapeutic consequence for the participating children follows the study participation. This particular type of research might provide important information for the future (8–10). However, in this type of research, it is even more important to tailor the research to children and develop a child-friendly approach since there is no direct benefit for the participating child (10, 11). Possible complaints from children are physical (such as pain and unpleasantness) and/or mental discomforts (such as anxiety, worries, and boredom) and should be taken into consideration (12). Some non-invasive procedures, such as assessment of sexual development, could be perceived as burdensome as invasive procedures (e.g., venipuncture) (13).

Our aim is to provide an example of different strategies that we used during the EFFECTOR-study to optimize participation and minimalize physical and mental discomfort experienced by the children (14). These are a collection of some tips and tricks and examples; cheap and accessible to apply in a wide range of studies.

## Method

The EFFECTOR-study is a long-term follow-up study of the offspring of different maternal cohort studies. It is designed as a cross-sectional cohort study (14). Ethical approval was obtained from the Ethics Committee UZ Brussels and the Ethics Committee UZ Leuven/KU Leuven and was registered at ClinicalTrials.gov (NCT02992106). A written informed consent was obtained from the parents. A total of 143 children, aged 4 to 11 years old, were included between June 2017 and March 2019. 294 eligible subjects were contacted by mail and subsequently by phone. One hundred and seven children were lost- to-follow-up because of changed postal address or phone number (5 to 11 years gap between original and follow-up study). From the 187 remaining subjects another 44 parents refused to participate. Resulting in an overall participation rate of 143 out of 294 eligible study subjects (48%) or 143 out of 187 subjects reached by phone (76.5%). Our study population included a negligible amount of children from ethnic minorities.

Data collection was performed as a single study visit preferably at home, but sometimes in a hospital environment as well, combining both invasive and non-invasive procedures. Since the study is a follow-up study after an average of at least 5 years after the original study in the mothers and because the study subjects are now the children instead of the mothers, a lot of attention has been paid to the consent-process and minimizing discomfort for the children. We did not use a standardized assessment to measure the effect of our interventions since the design of the themed changes was a gradual process.

We chose “superheroes” as the theme for the study because of the wide age-span of the study participants (4 to 11 years old). This theme provides many possibilities and both younger and older children know superheroes to identify themselves with. The imagination of the children is stimulated and the theme serves as an easy conversation starter.

## Results

### Before the Study/Home Visit

#### Inclusion Process

Before obtaining parental informed consent, the mothers were contacted through an information booklet they received by mail. After the parents procured this booklet, they were contacted by phone according to study protocol (14). A tailored approach was used for each phone call and when necessary, the parents could receive Supplementary material before giving consent to participate in the study. This material could be used by the parents in order to guide the children through their decision-making process.

#### Informed Consent and Assent

**Figure 1:**
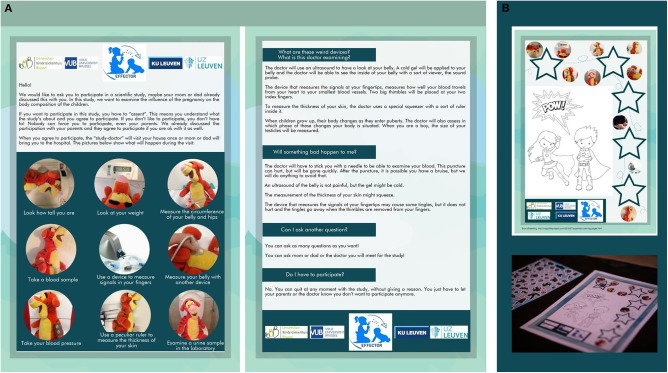
**(A)** This figure shows a translated version of the assent in English. The assent was provided on paper or electronically (A4 recto verso). **(B)** This figure shows the sticker diploma that we used during the study visit.

The informed consent for the parents was supplemented with a visually attractive assent, providing the study information tailored to children of primary school level (Figure 1A). The procedures were illustrated using photographs of a teddy bear, making it also useful for preschoolers. No graphical designer was used for the development of the tools, just an easy online design software. The photographs were re-used on the sticker diploma, which resulted in a sense of recognition for the children. The assent was sent to the parents before the study visit; it was also used the day of the study visit to check whether the children understood all the study procedures correctly.

#### Video Material

A Youtube video in superhero theme was developed, shot with a normal camera and edited by a hobbyist (Supplementary Video 1). All participating children could watch it through a private link. This video featured the study doctor the children would meet for the study visit, which was a nice way to break the ice once they actually met the doctor. Watching the doctor act crazy during a Youtube movie was considered cool and some of the participants watched the video repeatedly.

Supplementary to the funny superhero movie, the parents could obtain realistic video fragments of the performed measurements during the home visit (Supplementary Video 2). The necessity of this supplementary material was always discussed with the parents before the study visit. In a single severe case of autism spectrum disorder a “test-run” was done before the actual study visit to show the devices, meet with the study doctor and explain once more what was going to happen during the actual measurements.

### During the Study/Home Visit

The study visit consisted of many different procedures, both invasive and non-invasive (Supplementary Table 1). The sequence of the different tests was always identical in order to limit time-fluctuations as much as possible and keep the attention of the children. The average duration of one study/home visit was 60 to 90 min. All communication during the home visit by the study doctor was framed in the superhero theme. This communication was tailored to the age of the children and specific fears, when mentioned by the parents before the home visit.

At the beginning of each study visit, the assent was used to see whether the children understood everything and if there were specific study procedures they dissented. Before starting the procedures, appointments were made with the children. They were always offered the option if there were specific study procedures to which they dissented.

Children could choose to fill a sticker diploma, featuring the same photographs as seen on the assent. The participants were always free to decide whether they wanted to make a sticker diploma or not and could chose a wide range of different stickers (Figure 1B). Other used distraction methods were television or Netflix® videos when compatible with the performed tests. For the venipunctures, an additional local anesthetic was used (Lidocaine and Tetracaine).

**Figure 2:**
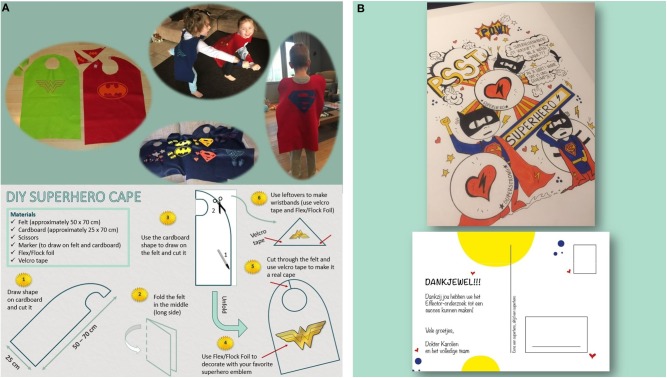
**(A)** This figure shows the super hero capes that we crafted and a DIY pattern to make them (written parental consent obtained for publication). **(B)** This figure shows the personalized post card the participants of the study received at the end of the data collection.

For the preschoolers, an extra feature was added by crafting superhero capes the children could use during the tests and keep afterwards as a reminder of their study participation. The superhero capes were low budget made with the use of felt (Figure 2A). Apart from the capes and the diploma, the children could also choose a small gift at the end of the visit (e.g., headphones, notebook, reading book, pen…). Children refusing certain measurements were also given the gifts.

### After the Study/Home Visit

A tailored postcard was designed for our study and all participants received a postcard after finishing the data collection process (Figure 2B).

We did not receive any negative feedback from parents of participating children. We did receive positive and spontaneous feedback from both parents and participants:

- “The doctor is funny and nice. I experienced less pain than expected. It's nice to know that you are helping other children when participating” (Girl, aged 11).
- “X really adores his cape, he didn't want to take it off for the rest of the day!” (Mother of a boy, aged 5).
- “The communication of the study is entirely in superhero theme and adapted to the child's age. As a parent you let your child participate in good conscience” (Mother of a girl, aged 11).
- “You really left an impression! She couldn't keep silent about it afterwards and she wants to be a superhero-doctor when she grows up” (Mother of a girl, aged 4).

We did not receive any negative feedback from parents of participating children.

## Discussion

Both mental and physical discomforts have been reported by children participating in scientific research. We presented a set of tools we used during the non-therapeutic EFFECTOR-study to make the study visit as child friendly as possible with a limited budget.

As suggested by children in qualitative research, they are more willing to participate when they receive age-appropriate information, when there is distraction during the procedures and when they receive a sign of appreciation after their participation (12). We illustrated that many of these suggestions are easy to implement in a study visit. The illustrated assent and Supplementary Video material proved to be very useful and handy for children of a wide age-span. Multimedia techniques are indeed useful to complement the paper-based information in the decision making process of the parents and their children (15, 16).

In our opinion, the assent is an indispensable addition to the informed consent signed by the parents, especially in non-therapeutic research (2, 17). The assent should focus on providing clear information about the procedures using comprehensible language since we assume children below 12 cannot yet fully grasp the content of medical procedures they never underwent (13).

We had a satisfactory overall participation rate of almost 48%; taken into account a large amount of lost-to-follow-up and a participation rate of 74% of those parents who were actually reached. Comparing participation rates is difficult, especially with our particular non-therapeutic and cross-sectional cohort study design. A comparable non-therapeutic study performed in Denmark in 2006, aiming at children aged between 6 and 16 years old, showed a participation rate of 62% for undergoing a venipuncture, urine sample and clinical examination (18). The researchers did not find socio-economical differences between assenting and dissenting children. The latter group reported more worries about the invasive procedures and fewer of them underwent a venipuncture in the year preceding the study (18). In a hypothetical thought experiment, a majority of parents and children would be willing to participate in non-therapeutic research, especially when the risks were compared to daily life risks (9).

When we compare the participation in our study to non-therapeutic studies in the same research field, our participation rate is situated in the upper half. A study on parent's perspective on child obesity cut-offs resulted in a response rate of 15% for a single questionnaire (19). A cross-sectional study on childhood obesity with anthropometric measurements in addition to questionnaires had a response rate of 37% (20). There are of course studies with higher participation rates, such as a long-term follow-up study on media consumption and the influence of the BMI of the children with rates ranging from 67 to 72% at different time points combined with questionnaires and anthropometric measurements (21).

Another entity of non-therapeutic studies are the (epi) genetic birth cohort and epidemiologic follow-up studies. The response rates in these types of long-term follow-up studies have been dropping during the last decades from 90% participation to below 70% participation because of a general decline in “volunteerism” and an anticipated burden to the hectic daily life of the twenty-first century (22, 23).

The main limitation to report is that we did not ask formal feedback from all the study participants and their parents. We judged this might feel as a burden for many of our parents since the study visit already took 90 min (with an average of 30 min of questionnaires beforehand). For our next follow-up studies, we will definitely use the superhero theme again and then use a short feedback possibility to make the results more tangible.

### Our Tips and Tricks

- Choose an overall theme that appeals to children of various ages.
- Provide visually attractive study documents. You do not need a graphic designer or a lot of money to design these documents! We used the online designing tool Piktochart.
- Use of a videoclip has definitely been an added value!
- Pay attention to the feedback of the children and their parents, especially in the beginning of your data collection process.
- There are a lot of simple and cheap do-it-yourself-tricks you can apply to change the children's experiences in your research.

## Conclusion

In spite of not offering a direct benefit to the participating children, non-therapeutic research should be continued in order to optimize future pediatric care. All efforts possible should be made to minimize mental and physical discomfort for the children. Framing in a specific theme is an affordable tool, easy to apply on all study material.

## Data Availability Statement

The data that support the findings of this study are available from the corresponding author upon reasonable request in case of a relevant IPD study.

## Ethics Statement

The studies involving human participants were reviewed and approved by the central Ethics Committee UZ Brussels and the local Ethics Committee UZ Leuven/KU Leuven (ClinicalTrials.gov NCT02992106). Written informed consent to participate in this study was provided by the participants' legal guardian/next of kin. Written informed consent was obtained from the minor(s)' legal guardian/next of kin for the publication of any potentially identifiable images or data included in this article.

## Author Contributions

KV, RD, and IG conceptualized and designed the study, drafted the initial manuscript, and reviewed and revised the manuscript. All authors approved the final manuscript as submitted and agree to be accountable for all aspects of the work.

### Conflict of Interest

The authors declare that the research was conducted in the absence of any commercial or financial relationships that could be construed as a potential conflict of interest.
