# Supplementary material for: How to Maximize Children's Involvement in Non-therapeutic Research—Lessons Learnt From EFFECTOR
Source: Front Pediatr. 2020 Feb 14;8:47. doi: 10.3389/fped.2020.00047 (PMC7040477; doi:10.3389/fped.2020.00047)
Supplement: Supplementary file 1 [file Table_1.DOCX]

**Supplementary Table 1**: This table provides an overview of the performed measurements during the home visit in the order of performance. For each of the procedures the used distraction methods are listed when applicable.

| Measurement | Used distraction |
| --- | --- |
| Measurement of height | No specific distraction used |
| Measurement of weight and body composition by bio-electrical impedance analysis (BIA)^1^ | No specific distraction used |
| Measurement of blood pressure | No specific distraction used |
| EndoPAT® assessment of endothelial function (peripheral arterial tonometry)^2^ | Television program or Netflix® video |
| Skin fold measurements and circumference measurements | Television program or Netflix® video |
| Ultrasound measurements | Television program or Netflix® video |
| Venipuncture | Television program or Netflix® video  Rapydan® local anesthetic |

*Footnotes:*

*1. For the bio-electrical impedance analysis a Tanita MC-780U was used. The child needs to step onto a scale and hold two handgrips. The device uses a low-level electrical signal from footplate and hand electrodes through the body. The measurement itself takes less than a minute.*

*2. For the measurement of the pulse wave amplitude, by using a finger plethysmograph, both index fingers of the patient are placed in pneumatic probes. First, the device performs 5 min of baseline measurement and thereafter a blood pressure cuff occludes the arterial flow of the arm for 5 min. After a rapid deflation of the cuff, a reactive hyperaemia takes place, which is a measure for the arterial endothelial function. The arterial occlusion can cause a tingling feeling in the occluded arm that disappears after deflation of the cuff.*
