# Supplementary material for: How to Maximize Children's Involvement in Non-therapeutic Research—Lessons Learnt From EFFECTOR
Source: Front Pediatr. 2020 Feb 14;8:47. doi: 10.3389/fped.2020.00047 (PMC7040477; doi:10.3389/fped.2020.00047)
Supplement: Supplementary file 4 [file Data_Sheet_1.DOCX]

Supplementary Material

# Supplementary Figures and Tables

## Supplementary Videos


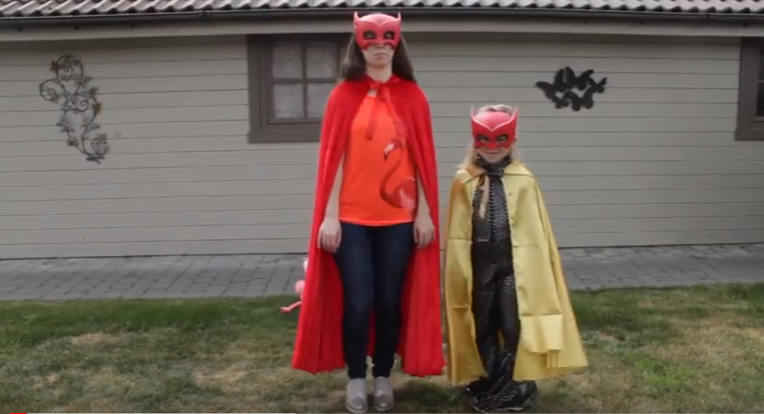


**Supplementary Video 1 (Superhero movie).** This video is the superhero movie that was shared through a private link with the parents before the study visit. (written parental consent obtained for publication)


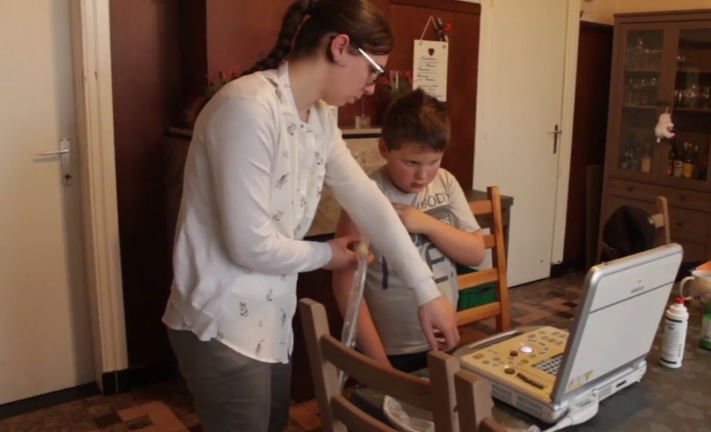


**Supplementary Video 2 (Realistic Video fragments).** This video material is realistic material that was used as supplementary material when parents thought it was necessary to provide their children with extra preparation for the study visit. (written parental consent obtained for publication)

## Supplementary Tables.

**Supplementary Table 1.** This table provides an overview of the performed measurements during the home visit in the order of performance. For each of the procedures the used distraction methods are listed when applicable.
